# “It’s a Big Family Here.” Becoming and Belonging in a Service Providing Employment-Related Support for People with Mental Health Problems: An Interpretative Phenomenological Analysis

**DOI:** 10.1007/s10597-021-00819-4

**Published:** 2021-04-09

**Authors:** Nisha Chauhan, Dawn Leeming, John Wattis

**Affiliations:** University of Huddersfield, Queensgate, Huddersfield, HD1 3DH UK

**Keywords:** Mental health, Supported employment, Interpretative phenomenological analysis, Lived experience

## Abstract

The impact of employment for individuals with mental health problems is complex. However, research suggests that when support is provided for accessing employment and gaining roles and skills that are valued by others, a positive effect can be seen on recovery. Employment-related support can take many forms and there is a need for further research into the experience of accessing different kinds of services. The current paper examines the lived experience of 11 people participating in a UK social enterprise providing work experience, training, and skills development for those with mental health problems. Although ‘sheltered’, the organisational ethos strongly emphasised service-user empowerment, co-production, equality with staff, provision of valued social roles and person-centred support. Phenomenological analysis revealed that participants valued a sense of belonging and authentic relationships within the service, whilst being given the opportunity to rediscover an identity that may have been lost because of their mental health problem. However, participants also discussed how, although the service improved their self-value, some feared the ‘real world’ outside of the service and were unsure whether they would be met with the same support. Tensions between field dominant approaches in supported employment and the experiences and values of the participants are explored. We argue that the findings highlight the importance of a nurturing working environment and the value for recovery of a range of meaningful roles, beyond competitive employment.

## Introduction

Individuals with mental health problems remain under-represented within the workplace, yet employment may play an important role in recovery, offering a sense of purpose, social inclusion, and value (Prior et al., 2013; Walsh & Tickle, 2013). Within the US and UK, the Americans with Disabilities Act (1990) and the Equality Act (2010) require employers to cater for employees with mental health problems. However, there is evidence that employers can be reluctant to hire individuals with mental health problems, fearing that they may not be able to meet the expectations of the role or that workplace stressors may cause relapse and increased workload for others (Bonfils et al., 2017; Fossey & Harvey, 2010; Goldberg et al., 2005; Olesen et al., 2013), particularly if employees have long term mental health problems. When they do employ individuals with mental health problems, employers do not always provide adequate support and flexibility (Brohan et al., 2012; Henderson et al., 2013). This is important to facilitate inclusion, as some working conditions can be detrimental to mental health and well-being (Burton et al., 2008; Knudsen et al., 2013). With good support, the experience of being in the workplace can increase self-worth, independence, and social inclusion, and provide a sense of purpose and normality (Burton et al., 2008; Drake et al., 2012; Litchfield et al., 2016; Nieminen et al., 2012; Prior et al., 2013; Slade et al., 2014). Employment seems especially helpful for recovery if it holds meaning for the employee (Doroud et al., 2015; Walsh & Tickle, 2013).

Recognition that work can promote recovery and that there is a need for employment that is sensitive to the needs of people with mental health issues has led to various interventions. Schemes offering individualised support to people with mental health difficulties to enter open employment directly without relying on pre-vocational training (Individual Placement and Support—IPS) have become increasingly popular. They have been shown to improve employment rates considerably (Brinchmann et al., 2020; Drake et al., 2012), challenging negative expectations about the employment prospects of people with psychiatric disabilities. However, it is less clear from a meta-analysis of RCTs that IPS supports improved mental health as effectively as it supports improved employment outcomes (Frederick & VanderWeele, 2019). Several authors have noted difficulties implementing IPS (e.g. Boardman & Rinaldi, 2013; Bonfils et al., 2017), and this may relate to Essen’s (2012) argument, based on careful analysis of surveys, that the desire for regular paid employment amongst those with long-term mental health problems may have been overstated when promoting IPS. Some people may prefer alternative approaches to returning to meaningful occupation. Other employment-support approaches use skills-training and sheltered working alongside open employment or as steppingstones to this (Yu et al., 2016), though there has been longstanding concern about the segregated nature of sheltered work (Migliore et al., 2007) and thus the potential to make service users feel that they are defined by their mental health difficulties (Koletsi et al., 2009) and foster dependent relationships (Tangvald-Pederson & Bongaardt, 2017). A few small-scale studies have confirmed that people with mental health difficulties value a range of employment support schemes (De Malmanche & Robertson, 2015; Olney & Emery-Flores, 2016; Yu et al., 2016). However, research is still limited (Olney & Emery-Flores, 2016). Further studies are important for understanding the wide variety of approaches to facilitating meaningful work experiences and valued roles for those with mental health difficulties, and the way in which particular employment support service structures and cultures can shape the experience of individual service users. This is particularly important given that some work cultures can be harmful and devaluing (Koletsi et al., 2009) and that the ethos of an employment support scheme may be as important as the specific services it offers.

The research reported here used a phenomenological approach to explore in depth the lived experience of taking part in a service that provided work experience, training, and skills development to people with mental health problems. The employment service was integrated into a larger social enterprise providing a broad range of mental health care services. Although the setting was relatively sheltered, there was a strong emphasis across the social enterprise on service-user empowerment, co-production, equality with staff, provision of valued social roles in a commercially viable setting, community engagement and person-centred support. The findings may be relevant to others considering whether to develop services along similar lines.

## Methods

### Interpretative Phenomenological Analysis

Given the emphasis of the service on co-producing a service culture that fostered confidence and a sense of inclusion, it was important not only to capture service-user perspectives on tangible aspects of service provision, but also to explore service users’ lived experience of participation, and the subtle meanings of this for them. Phenomenology was an ideal fit as it is concerned with understanding our conscious experience in the world from a first-person point of view (Gallagher, 2012). Using the idiographic focus of Interpretative Phenomenological Analysis (Smith et al. 2009) enabled exploration of whether and how the service achieved its aim of person-centred support. IPA draws on both descriptive and interpretative approaches to phenomenology, aiming to report experience, such as engaging with training and becoming part of a work team, as it appears in consciousness, but also accepting Heidegger’s view that interpretation is inevitably part of experience, and part of the researcher’s developing understanding of that experience (Mjosund et al., 2017). The analyst attempts to understand and describe the participant’s experience of being-in-the-world, with a focus on particular instances of lived experience (Larkin et al., 2006), whilst exploring what it means to that person in that context, through interpretative analysis (Finlay, 2008). The analysis may also draw cautiously and reflexively on theoretical concepts in keeping with phenomenology’s understanding of person-in-context, provided these concepts illuminate participants’ meanings and experiences, rather than imposing predetermined meaning (Smith et al., 2009).

### The Employment Service

This study was conducted in a comprehensive service for adults with severe mental health problems in England run by a ‘not-for-profit’ social enterprise, where provision of employment support was integrated with broader mental health care. The service was inspired by the principles of person-centred working (McCormack & McCance, 2016) and social role valorisation (Wolfensberger, 1983). The whole organisation focused on treating and supporting people with severe mental health problems, often undervalued by society, and facilitating a better sense of their own worth and value. It’s guiding principles included the idea that there should be a partnership between staff and those they worked with, and that all should be regarded primarily as people rather than clients, patients or even ‘service users.’ Illustrations of this were the limits placed on ‘staff-only’ spaces and the involvement of service users in staff training, service design and staff recruitment. Service principles also included creating valued roles for people, fostering a sense of community which encouraged continued involvement of service-users between episodes of mental health crisis, and involving the wider community to promote better understanding of mental health. The employment scheme was part of this organisation, though to some extent it had predated and been used as a model for the whole social enterprise. Unlike many sheltered work experience schemes, this service provided members with the opportunity to interact with the public in normalising ways, for example in activities as diverse as running an off-site public-facing, commercially viable snack bar and retail garden centre as well as providing all catering, portering, maintenance and cleaning services to inpatient and outpatient facilities and staff. An overriding aim was to support service users to develop meaningful and valued social roles, both within the service and, by enhancing employability skills, in their future lives. Service users could hold the title of member and/or volunteer, all of whom (service users, carers, or staff) had equal voting on senior appointments and policy. They could also be voted onto the operational board. Those using the service were given support and guidance whilst gaining experience in their field of work, including opportunities for qualifications and training in basic employment skills such as numeracy and literacy. Volunteers, who had often been long term users of the employment support service, had additional responsibilities such as managing members in their field of work, or peer mentoring. A significant number progressed to paid employment within the service.

### Participants and Methods

The aim of IPA is a detailed and immersive exploration of individual experience and meaning making, and therefore a small sample of participants is used (Willig, 2008). A relatively homogenous sample is often used, in order to explore a particular experience in a particular context, though the extent of homogeneity varies according to practicalities and study aims (Smith et al., 2009). In the present study, the emphasis was placed on shared experience of the culture of a particular service, rather than homogeneity with regard to socio-cultural factors or previous experiences. Eleven users of the employment service took part—three men and eight women, aged 21–60 years of age. Detailed characteristics of the sample were collected as advised by Rose et al. (2019) to allow for transparency in the analysis. Two had undergraduate degrees, and two had A-level qualifications (UK qualification at age 18 used for University entry). Seven had previously held full time jobs, two had held part time jobs, and two had not worked before. Each participant had experienced a mental health problem for at least 2 years, where diagnoses included bipolar disorder, schizophrenia, depression, and anxiety. All lived independently, or with their parents, families, or partners. Most participants were members of the organisation, whilst two had progressed from member to volunteer or paid staff member. Most participants were also in receipt of welfare payments [Enhanced employment and support allowance (EESA), and personal independence payment (PIP)]. Reflecting the predominant demographic characteristics of the users of the wider mental health services and the local community, all were White British. Through a semi-structured interview lasting approximately half an hour, participants were encouraged to discuss a variety of topics: how they came to join the service; how life had changed since becoming a member; what influenced their decision to work in a particular area within the service; what their experience of the service had been and their relationships with other service-users and staff; moments where the service had impacted on their lives, if any; therapeutic effects of the service; the ethos of the service and the sense they made of this. They were also given an opportunity to discuss anything else about their experience within the service. Each interview was transcribed, with any identifying information replaced with pseudonyms or omitted. The data was analysed using IPA’s approach of reading and re-reading the data for each individual several times to develop in-depth understanding of that individual’s experience (Willig, 2008). After this, exploratory descriptive and linguistic comments were made. Time was taken to understand what mattered to the participant within their experiences, and how their use of language demonstrated this. Following this, emergent themes were developed (Mjosund et al., 2017). This process was repeated for each transcript. Careful consideration was given to individual experience by attempting to place on one side analysis of the previous transcript when interpreting the next (Bishop & Shepherd, 2011; Smith & Osborn, 2007). After this, patterns were identified across all the participants’ interviews, and a reconfiguration and re-labelling of themes was carried out (Mjosund et al., 2017).

### Compliance with Ethical Standards

Approval was granted by two ethics committees: A University Research Ethics Panel, and the relevant National Health Service (NHS) Research Ethics Committee. Informed consent was obtained from all individual participants included in the study with the right to withdraw at any point before data analysis. Interviews were recorded, anonymity was ensured, and data were held securely in accordance with University policy.

The study received no external funding. The authors declare that they have no conflict of interest. All authors certify their responsibility for the manuscript.

## Findings

Three superordinate themes were identified: *Becoming the Best Me; Acceptance and Belonging* and, *Safety or ‘Not a Real Job’?* These are presented in turn below, with sub-headings indicating subthemes within each superordinate theme.

### Becoming the Best Me

In most interviews there was strong engagement with a narrative of personal development, as participants explained how, through involvement in the service, they had come to view themselves more positively. Several indicated that the service had enabled them to recognise skills and abilities they had not previously been aware of.

#### Participating in the Service Makes Me now Feel more Positive About Myself

Working as a valued member of a team positively impacted participants’ self-perception. When praised for their work, participants felt they were being noticed for positive attributes in a way that they had not been previously. This, in turn, increased their self-worth:

‘They get me to do something new, challenging me. It makes me feel good about myself… they praise you when you leave the shift, they’re always putting notes on the board, saying thanks for the staff.’ (Kate)

Some reported that the tasks they were assigned in the service allowed them to have more responsibility than they had experienced in the past, making them feel more valued and positive about themselves. For example, some were offered a role helping others who were struggling:

‘[…] my confidence has really built, and I’m pleased of where I am now, because I’ve helped supporting the learners in class. It gives me such a sense of achievement. It really does. And seeing them progress is even more amazing.’ (Claire)

Like many, after a long battle with education, Claire had the skills to help others who had been in similar situations as herself. Succeeding in tasks was not the only factor making participants feel more positive about themselves—appreciation from others was important:

‘I love the garden centre and work up there. Richard’s proud of it, and I am too. Everyone’s proud of it.’ (Lewis)

Praise, taking responsibility and succeeding, enabled participants to develop more positive views about themselves.

#### Becoming who I Ought to be

Participants discussed how the service learned about participants and allowed them to recognise who they really were, by avoiding focusing on their mental health issues. Participants could learn and work in areas of existing interest, discovering a person within that they felt had always been present. The service also helped the participants find new interests, and they found they flourished in areas they had not expected, and learned about a person they did not know existed within them:

‘I feel good, that I can do it. I never used to bake, but now I can. Half the stuff I do in there, I never knew I could do it.’ (Jane)

‘I’m always learning new things, things I thought I couldn’t do and I can.’ (Ella)

Given the chance, participants found skills that they had not used yet. This did not just include finding skills in new areas, but in areas where they had struggled in the past, such as in education:

‘I always thought I was thick, I got bullied, I got told I was thick, oh you’re stupid, you’re an idiot. And I started believing it. But I know it’s because I’m dyslexic. […] I do Maths and English here.’ (John)

Participants were assessed for various disabilities, including dyslexia. For some, this was an explanation of past struggles in education where disabilities had not been recognised. With this discovery, they were able to flourish in difficult subjects with support.

Contact with the service helped participants move towards becoming the person they felt they ought to be. However, for Kate, contact with mental health services was problematic, as there were aspects of this that she struggled with, such as her diagnosis:

‘And to accept the diagnosis, umm, what I was given. I do and I don’t accept it. I accept because it is a label, but I don’t want to accept it into my life, so I can still have the freedom of movement.’ (Kate)

Kate had not wanted to accept her diagnosis because of the limitations she believed it would bring. For her, engaging with mental health services raised the possibility of a negative identity which needed active management. However, for most participants, contact with the service enabled them to discover positive aspects of themselves and positive identities that had been hidden or denied. For some, this meant being able to take pride in demonstrating the skills they knew they had; for others it was about learning new skills. For some, such as Jane, Ella and John, there was a process of uncovering something they felt they ought to have always known about themselves.

### Acceptance and Belonging

All participants noted the positive relationships within the service, and most made some reference to a sense of belonging. This was related to feeling valued by a group of others with whom they felt some commonality and with whom they experienced authentic relationships. Often this contrasted with previous experiences of feeling marginalised and alone. Positive responses from others seemed key for the personal and skills development discussed above. These ideas are explored via three subthemes:

#### Being with People Like me

All participants noted and valued a sense of equality between all in the service. Uncertainty as to who was staff and who was a member strengthened this sense of equality and acceptance:

‘Some people, you wouldn’t even know, that they’re actually a member, and you would think were staff.’ (Robin)

‘When you walk in, you can’t tell who is staff and who is a member […] no power-dressing, that’s the ethos. I feel valued – I can’t explain, it’s amazing.’ (Alice)

The knowledge that others in the service also shared a mental health problem offered reassurance and feelings of being understood, as Ruth explained:

‘I still get embarrassed here in Jeff’s class, but I know they’re there to help and I apologise because they know where I’m coming from and don’t hold it against me.’ (Ruth)

Participants felt others in the service were more than work colleagues—there were feelings of mutual support and a sense of unity amongst participants and other members of the service. These feelings allowed them to feel at ease with one another and several referred to other service members as ‘friends’ or even ‘family’:

‘I enjoy it, the atmosphere, and the people that work in the kitchen, just friends. We just click. When it’s hard, they all help each other. You can have a laugh and a joke with everyone.’ (Jane)

‘It’s a big family here. The atmosphere is great here.’ (John)

When problems did arise, members were able to support one another through their shared experiences. Several echoed Ruth’s explanation of ‘they know where I’m coming from’. This atmosphere made it easier to relax with one another and be who they really were:

‘I was nervous at first, but I think the open attitude and the banter, that’s what broke the ice. Because it allowed me to let go of my anxieties and trust, and build a bond well, essentially banter.’ (Tom)

‘But I love talking to them [service users who are customers], I know where they’ve been. I feel something when I come here, I feel like a sense of belonging, that I belong here. I haven’t felt that any other place before. I don’t know why, I think it might be the ethos of the place really.’ (Alice)

The openness of others in the service ‘broke the ice’ whilst shared experiences increased participants’ feelings of belonging.

#### Building Authentic Relationships

Participants also valued people’s honesty and not being treated as if they were fragile or childlike because of their mental health issues. Several commented further on the open, honest, and authentic relationships in the service. They did not fear judgement, as they knew the open nature of the service could not allow it, and everyone was clear about what they thought. Claire, who has two prostheses, explained this after an incident in the classroom:

‘I remember doing my first English class with Jeff, and I was about to stand up and all of a sudden, my [prostheses] fell off (laughs)! Well, I just laughed, and Jeff laughed. One of the learners got angry, and said, ‘You can’t laugh at Claire! It’s not funny!’ But it’s the best thing he could have done. I didn’t care, and I will talk to anybody about it.’ (Claire)

This openness is something that many participants admired:

‘I still distrust strangers…. I found people attached to [the service] are indeed more open than those in the streets.’ (Tom)

Tom also found it helpful that a staff member did not feel the need to agree with him:

Interviewer: [After Tom says he’s always been looking for acceptance] ‘Do you feel like you’ve been accepted here?’

Tom: ‘Yes, what I saw was open minded people, and that’s what I need, …. who can openly say I think you’re right, I think you’re wrong. … It’s like, one of the staff members at the gardens (catering facility)…He’s like, I need proof […] I say if it’s white, why is it not black, or something? It’s the kind of person he is, and I like that kind of argument.’ (Tom)

Tom seemed to value that the staff member did not appease him by falsely agreeing but maintained his authenticity. He interpreted this as a sign of being accepted. A few other participants also indicated that members were not treated as fragile individuals who needed to be sheltered, but as adults:

‘They don’t treat you like a child here. It’s a job. That’s what the Job Centre [a government funded employment agency] didn’t know.’ (Lewis)

Whilst understanding and accepting each other’s mental health problems, those in the service were prepared to challenge participants. For Claire, her low mood was preventing her from trying courses in the service:

‘Jeff always tells me you’re not thick, you can do it, you can do it. Even when I told him to fuck off! On numerous occasions (laughs). But he gave me that kick that I needed, and never got angry. He gave me a kick start. He was fantastic. He was amazing.’ (Claire)

Jeff’s persistence resulted in Claire trying a course, and finding she could succeed when supported, whilst learning that Jeff genuinely wanted the best for her.

#### A Service Which Wants Me Here

The feeling of being wanted and needed allowed participants to have positive experiences. Often this was a contrast with previous experience and the different approach of the service made them feel valued:

‘I hated it there [The Job Centre]. They made me feel like a nobody. You had to fill in forms, and I hated it. No one helped me do them, they said you can’t do that, I had to do it myself. Here, somebody will help me.’ (Ella)

Participants discussed how this made them feel the service was concerned about their welfare, rather than just completing their job role, as Robin experienced during a period as an inpatient when unwell whilst working with the employment service:

‘[The service] was brilliant, even at the time, Grant, the chief exec came to see me on the ward. They made you feel, like, you know, in any business it is a business, but they made you feel valued and that you weren’t just a number.’ (Robin)

Robin appeared amazed that the chief executive of the service was concerned about her, epitomising what participants felt about the service.

This theme of *acceptance and belonging* was noticeable across the data and stands in sharp contrast to several participants’ accounts of previous stigma and discrimination in other situations. The accepting relationships with others in the service seemed to have been crucial for developing a sense of purpose, gaining work-related skills, and becoming the person they wanted to be:

‘[The service] gave me structure, something to get out of bed for, that I knew there was someone waiting for me to go in, and that I was wanted and needed and of use. That I knew I would get my skills back, umm. They’ve built my confidence, I felt included.’ (Lewis)

Without the nurturing relationships within the service, participants felt they would not have been able to realise their potential and, as Lewis suggests, feeling needed and that others within a team relied on them seemed particularly important.

### Safety or ‘Not a Real Job’?

Many of the participants talked about the service as a safe and protected environment. Some saw this as a valuable stepping-stone to mainstream employment:

‘Yes, it was a bubble, and you’re in a protected environment, but it was essential to go back into normal life. I was there for 2 years, and then became a volunteer.’ (Robin)

However, for others, there was continuing concern regarding the unpaid nature of the work, the disadvantages of this and consequent concerns for the future.

#### I am not Being Paid

Many participants reported a sense of the service as temporary, not a direct route to future employment, and that they would inevitably need to move on. Much of this was due to financial issues and commitments outside of the service. Some feared their mental health might suffer in the long run:

‘But there’s time where things get on top of me, that I’m not earning any money from it, and I’m scared of where I might end up.’ (John)

Most were aware of financial strain due to a lack of paid employment and knew this could not be permanent. John went on to explain his feeling that the work he did within the service was not a real job *because* he was not being paid, though he expresses ambivalence about this:

‘Well, sometimes I think I’m doing all of this for nothing. It just seems sort of pointless sometimes, and it makes it hard. But with [the service] there’s something about it, and I don’t mind doing it for nothing, sure I’d love for it to be paid. I would love to work here properly. I would be able to relax and chill, and I’d have the support here and everything when I needed it. And you’re not judged for anything.’ (John)

As John suggests, concerns about lack of payment did not necessarily outweigh the positives and the value of support from the service. However, the feeling that the service did not offer a ‘real job’ was not just due to the lack of money *per se*, but the feeling of being under-valued because of not being paid:

‘I feel less discriminated against, the only thing is, because it’s not a real job, it’s voluntary, you haven’t got that ethos of being paid.’ (Kate)

Benefits were the only form of income for most of the participants, which had negative connotations:

‘That’s why I want a job, because I don’t want to be on benefits. I want to do it myself’ (Lewis)

Being reliant on benefits, rather than earning a wage, resulted for some in feelings of not being independent. They wanted to be able to use their own earnings to survive, which for many was not possible. Several, including Lewis, expressed mixed feelings about this, recognizing that being able to ‘give back’ as a volunteer seemed an important part of belonging to a valued community:

‘I don’t get paid here, but I feel important being a volunteer. I’m putting something back into the community. I do a lot of charity stuff for them... I am proud of it. Because they’ve done so much for me, I put something back. It’s a lovely organisation.’ (Lewis)

#### An Uncertainty for the Future

Although the service offered support and a safe place for many of the participants, they did not see this as a long-term solution. For many, there was still a sense of uncertainty for what the future held. Some were unsure whether they would be allowed to return to work at all:

‘I don’t know if I can go back to work, they haven’t given me a deadline on how soon I can work’. (Kate)

Others were afraid of the possibility of discrimination within open employment. This was not limited to prejudice about mental health problems, but other factors, such as age:

‘I’ve been applying but haven’t heard anything back from any of them. But what can you do. I always tick the box for mental health, but I think it’s my age that makes them not call me back’. (Jane)

Some felt that all the work they were doing might end with no employment prospects and barriers would always arise:

‘I keep seeing barriers. I know they say don’t look at them, and I just think but they just keep coming up. You get rid of one barrier, and another one, BOOM, turns up. And it happens again and again and again.’ (John)

#### Am I Safer in the Service than in the World Outside?

Many had negative work-related experiences in the past and, despite financial and other concerns, were therefore wary of leaving the service, fearing discrimination or a lack of support in future workplaces:

‘I think if I’m not here, what’s going to happen, will I get a job, will they be supportive, appreciative, giving praise like they do here?’ (John)

‘I still don’t feel well enough to go back to work, you know to go into mainstream employment again, where they would be no support like I get here... You get panic attacks, and you know, it becomes a physical problem too’. (Alice)

Several others also referred to not being ready to leave the service and this led to participants hoping for paid positions in the service (see previous quotation from John). This was ideal—a way to maintain both their current supportive environment and a ‘real’ job.

Moving on to paid employment within the service was possible for some, yet one participant who achieved this still noted the risk of becoming institutionalised, as if this might be a side effect of staying within a safe space:

‘I wasn’t quite ready to leave [the service]. And you do become institutionalised, you know, it’s a safety net, so it was a bit scary, but I knew that if I didn’t take that step [taking a paid position within the social enterprise, initially via an employability scheme]… I knew I might have to go to outside employment instead, which I wasn’t ready for and I didn’t want to.’ (Robin)

Some did not feel the service was safer than the outside world, including some who has been outpatients or inpatients within the wider mental health services, and those with previous positive employment experiences. For others, it appeared that the service was the last destination in their recovery, despite it aiming to help members gain paid employment. Ruth’s comment is a positive endorsement of the service but also indicates the difficulties of returning to mainstream employment: *‘I’d stay here as long as I can’.*

## Discussion

The findings suggested that developing work skills was only part of what participants valued. Many of the participants expressed positive views about the sense of community fostered by the service. A sense of belonging, where they experienced valued, authentic relationships enabled them to develop confidence and a new perspective on the skills they already had and those they developed. Therefore, the participants’ experiences could be seen as demonstrating how the service met many of the ‘Being, Belonging and Becoming’ occupational needs previously identified as important for those with mental health difficulties (Carter & Fuller, 2016; Rebeiro et al., 2018). The findings demonstrated how these needs were intertwined, so that participants redefined and rediscovered themselves (Being needs) through a sense of belonging and acceptance (Belonging needs) and skills development (Becoming needs). The ethos of the employment service seemed crucial, as development of the sense of being a valued worker reflected participants’ place in the group, the sense of equality in the service, and the feedback received from others. This is in keeping with symbolic interactionist understandings of identity development, drawing on Cooley’s early work on the ‘looking glass self’ (Doroud et al., 2018).

In the first superordinate theme, *becoming the best me*, the service provided valued roles for participants which they could be proud of and that engaged feelings of hope. Previous research has also shown how settings which provide such roles can enhance self-esteem and identity (Doroud et al., 2018). Participants in the current study discussed how the service allowed them to find their own individuality and feel much more positive about who they were. Previous experiences of stigma, shame and discrimination related to their mental health difficulties had led many participants to doubt their self-worth. Many mental health problems include negative views about the self at their core, leading to people struggling with a sense of shame or poor self-worth even before experiencing mental health stigma (Thornicroft, 2006). As a result, the need to promote self-worth and a sense of value to others is important. The interviews demonstrated that many of the relationships participants built within the service enhanced self-worth by aligning with the ‘I-Thou’ relationships promoted within person centred mental health care by Kitwood (1997), drawing on Buber (1958). Relationships were experienced as authentic and often supported participants’ perceptions of their worth (Buber, 1958; Kitwood, 1997; Lexen & Bejerholm, 2016). These relationships allowed participants to feel they belonged in a community, which valued their skills, as identified in the second superordinate theme, *acceptance and belonging.*

It is worth noting that, although open employment is now a key goal of many employment support services for people with mental health difficulties (e.g. Hutchinson, 2017), mainstream employment does not necessarily provide such a strong sense of community, acceptance and belonging as the participants appeared to have experienced within the employment service. In fact, few of them had experienced this previously. Prior research suggests that nurturing environments with supportive and accepting relationships have a positive effect on recovery (De Malmanche & Robertson, 2015; Doroud et al., 2018), yet there can be challenges in forming this kind of relationship within the workplace (Tangvald-Pederson & Bongaardt, 2017; Hodgson et al., 2019). The sense of belonging can be limited at first until acceptance has been received. This sense can become stronger as an individual chooses how they want to continue to belong in the workplace (Hodgson et al., 2019). Additionally, ambivalence can occur in wanting to be looked after and cared for, whilst also wanting professional appreciation (Tangvald-Pederson & Bongaardt, 2017), as indicated by the final superordinate theme in the present study, *safety or ‘not a real job?’.*

Despite many of the participants’ experiences of competitive employment being very different from the experience in the service, some expressed concerns that, by being protected, their present placement might be a less valued version of employment. Several were not confident that it was a steppingstone to ‘real’ valued employment, that is paid employment. The question for some was whether sheltered unpaid work did promote a socially valued role, in comparison to mainstream employment. Wolfensberger’s (1983) theory of social role valorisation (SRV) would suggest that real-world approaches, such as paid work, may often be more ‘normalised’ and valued and hence more beneficial to individuals with mental health problems within social contexts where paid employment is core to positive social identity. This is often the assumption of employment schemes which support people into mainstream competitive employment and, in fact, the limited research literature on ‘stepwise’ employment support in non-competitive or sheltered settings suggests this rarely leads to competitive employment and can often lead to clients becoming dependent on the vocational program (Drake et al., 2012). These were clearly concerns expressed by several of the participants and are a reminder that sheltered employment schemes may not always be the best option if re-entry to competitive employment is a client’s primary concern. However, it is also worth noting that occupational recovery is not necessarily a matter of paid employment, however important that may be for some people. Re-engagement with meaningful and fulfilling streams of occupation can include many forms of activity, not just formal employment (Rebeiro, 2001). Echoing the findings of previous qualitative research reviewed by Essen (2012), participants in the present study valued the skills they gained and the range of socially valued roles and connections with others that these skills afforded, regardless of whether skills development was a steppingstone to employment. Paid work was valued by many of the participants in the present study but not all, and this seemed to depend partly on the cultural meaning of paid work for them as well as their material circumstances. These findings are a reminder of the importance of listening to the perspective and personal recovery goals of service users, and that recovery needs to be ‘survivor-led’ as much as policy driven (Khan & Tracy, 2021). Assuming that one size of employment support is an appropriate fit for all seems unwise.

### Limitations of the Research

The current study focused on a small sample size within one service. This was advantageous for in-depth exploration of lived experiences of participation in a unique service which might inform service developments elsewhere. However, any attempt to generalise the findings would be inappropriate, and other users of the service may have had different experiences. Furthermore, service-users’ views and experiences do not necessarily accord with other data about recovery outcomes. Therefore, there is a need to continue research into varied employment support services, using different methods and perspectives in order to provide a fuller picture of the possible impacts of different kinds of employment support on recovery.

Another issue is that service users who are broadly positively disposed towards a service they feel an attachment to may be reluctant to mention negatives. In the current study, an attempt was made to reduce the likelihood of this bias by using an interviewer who was known to be independent of the service. However, a degree of positive bias cannot be discounted.

## Conclusion

This in-depth exploration of the experience of participating in a unique employment support service adds to the evidence base about the relative impacts of different kinds of services to support re-engagement with employment during recovery from mental health problems. The findings indicate that engagement with an employment scheme that, despite a relatively sheltered setting, promotes socially valued roles (e.g. with the local community and by mentoring others), alongside service-user empowerment, egalitarianism and positive, person-centred relationships can have many benefits that arise as much from the organisational ethos, as from the specific services offered. These include a sense of self-worth, skills-development, confidence, a sense of purpose and meaningful connection with others. In-depth exploration of participants’ experiences has revealed that debates within the literature about the setting and focus of employment support schemes may not always be capturing key concerns of service-users. What seemed to matter most to many of the present participants was the relational aspects of the experience—being authentically part of a group that valued them and being able to contribute in a meaningful way to the service that provided their mental health care. These less tangible aspects of the support received may have been fostered by the context—a not-for-profit social enterprise with the freedom to build decision-making mechanisms which actively involved service-users and that had striven to reduce distinctions between those with and without mental health difficulties. However, the findings also suggest that these benefits may not necessarily be sufficient for clients to feel prepared for transition to mainstream employment. Employment support schemes may need to pay additional attention to enabling this transition where it is desired. Previous research, and the accounts of participants in this study, suggest that participants’ caution about mainstream workplaces may not be entirely misplaced. Some workplaces can foster interactions and practices which are harmful to mental health (Gold et al., 2016) and worry about coping with the stresses of work can be a serious challenge for some individuals recovering from mental health difficulties (Essen, 2012). Therefore, employment or work experience in protected spaces may remain a preferred option for some. However, recognition of this does not negate the need for continued efforts to improve practices within mainstream employment settings to facilitate employee wellbeing and offer support to those with mental health difficulties who wish to benefit from the many positives of mainstream employment. Consideration of the relational factors that present participants valued but had not often experienced previously in mainstream work (expectations from others that they could achieve, authentic relationships, a sense that they mattered to others) provides pointers for what needs to change within some mainstream employment for it to be as inclusive as possible of people with mental health difficulties. This may also be healthier and less stressful for all. This suggests that, as well as promoting mental health awareness in mainstream workplace policies by including flexible working hours, occupational health counselling or modified job descriptions there could be greater emphasis on facilitating positive relationships and self-worth at work. Although this is already a consideration in many supported open employment schemes using IPS, the success of such schemes has often been measured by presence in the workplace, for example length or regularity of employment (e.g. Bond et al., 2020; Bonfils et al., 2017; Brinchmann et al., 2020), rather than an individual’s experience of a placement. However, the present study suggests that experiential aspects and work relationships may be vital for recovery. A recent study found that supported mainstream employment had the greatest impact on quality of life for those recovering from mental health problems when it included mechanisms for shared recognition of achievement and mutual support (Hodgson et al., 2019). The current findings, though based on a small sample, suggest not only that such relational factors may be important, but also that it is important to listen carefully to the perspectives of those in recovery about the socially valued roles they are seeking, and the kinds of environments in which they would prefer to develop these roles.
